# Exacerbation of cigarette smoke-induced pulmonary inflammation by *Staphylococcus aureus *Enterotoxin B in mice

**DOI:** 10.1186/1465-9921-12-69

**Published:** 2011-05-27

**Authors:** Wouter Huvenne, Ellen A Lanckacker, Olga Krysko, Ken R Bracke, Tine Demoor, Peter W Hellings, Guy G Brusselle, Guy F Joos, Claus Bachert, Tania Maes

**Affiliations:** 1 Upper Airways Research Laboratory (URL), ENT Department, Ghent University Hospital, Ghent University, Belgium; 2 Department of Respiratory Medicine, Ghent University Hospital and Ghent University, Ghent, Belgium; 3 Department of Pathology, Ghent University Hospital, Ghent University, Belgium; 4 Laboratory of Experimental Immunology, University Hospitals Leuven, Catholic University Leuven, Leuven, Belgium

## Abstract

**Background:**

Cigarette smoke (CS) is a major risk factor for the development of COPD. CS exposure is associated with an increased risk of bacterial colonization and respiratory tract infection, because of suppressed antibacterial activities of the immune system and delayed clearance of microbial agents from the lungs. Colonization with *Staphylococcus aureus *results in release of virulent enterotoxins, with superantigen activity which causes T cell activation.

**Objective:**

To study the effect of *Staphylococcus aureus *enterotoxin B (SEB) on CS-induced inflammation, in a mouse model of COPD.

**Methods:**

C57/Bl6 mice were exposed to CS or air for 4 weeks (5 cigarettes/exposure, 4x/day, 5 days/week). Endonasal SEB (10 μg/ml) or saline was concomitantly applied starting from week 3, on alternate days. 24 h after the last CS and SEB exposure, mice were sacrificed and bronchoalveolar lavage (BAL) fluid and lung tissue were collected.

**Results:**

Combined exposure to CS and SEB resulted in a raised number of lymphocytes and neutrophils in BAL, as well as increased numbers of CD8^+ ^T lymphocytes and granulocytes in lung tissue, compared to sole CS or SEB exposure. Moreover, concomitant CS/SEB exposure induced both IL-13 mRNA expression in lungs and goblet cell hyperplasia in the airway wall. In addition, combined CS/SEB exposure stimulated the formation of dense, organized aggregates of B- and T- lymphocytes in lungs, as well as significant higher CXCL-13 (protein, mRNA) and CCL19 (mRNA) levels in lungs.

**Conclusions:**

Combined CS and SEB exposure aggravates CS-induced inflammation in mice, suggesting that *Staphylococcus aureus *could influence the pathogenesis of COPD.

## Background

Cigarette smoking is associated with an increased risk of bacterial colonization and respiratory tract infection, because of suppressed antibacterial activities of the immune system and delayed clearance of microbial agents from the lungs [1]. This is particularly relevant in COPD patients, where bacterial colonization in the lower respiratory tract has been shown [2]. These bacteria are implicated both in stable COPD and during exacerbations, where most commonly pneumococci, *Haemophilus influenza, Moraxella catarrhalis *and *Staphylococcus aureus (S. aureus) *are found [3]. Interestingly, colonization with *S. aureus *may embody a major source of superantigens as a set of toxins are being produced including *S. aureus *enterotoxins (SAEs) [4]. These toxins activate up to 20% of all T cells in the body by binding the human leukocyte antigen (HLA) class II molecules on antigen-presenting cells (APCs) and specific V beta regions of the T cell receptor [5]. Between 50 and 80% of *S. aureus *isolates are positive for at least one superantigen gene, and close to 50% of these isolates show superantigen production and toxin activity [6].

During the last few years, it became increasingly clear that SAEs are known to modify airway disease [7], like allergic rhinitis [8], nasal polyposis [9] and asthma [10]. Furthermore, studies have shown a putative role for SAEs in patients suffering from the atopic eczema/dermatitis syndrome (AEDS), where colonization with *S. aureus *is found more frequently (80-100%) compared to healthy controls (5-30%) [11], and *S. aureus *isolates secrete identifiable enterotoxins like *Staphylococcus aureus *enterotoxin A and B (SEA, SEB) and toxic shock syndrome toxin (TSST)-1. Until now, evidence for SAE involvement in the pathogenesis of upper airway disease like chronic rhinosinusitis with nasal polyposis (CRSwNP), arises from the finding that IgE against SEA and SEB has been demonstrated in nasal polyps [12] and levels of SAE-specific IgE in nasal polyposis correlated with markers of eosinophil activation and recruitment [13]. Similarly, in COPD patients, a significantly elevated IgE to SAE was found, pointing to a possible disease modifying role in COPD, similar to that in severe asthma [14]. Moreover, we have recently demonstrated the pro-inflammatory effect of SEB on human nasal epithelial cells *in vitro*, resulting in augmented granulocyte migration and survival [15].

In murine research, the role of SAEs as inducer and modifier of disease has been demonstrated in models of airway disease [16,17], allergic asthma [18], atopic dermatitis [19] and food allergy [20]. These findings highlight the important pathological consequences of SAE exposure, as these superantigens not only cause massive T-cell stimulation, but also lead to activation of B-cells and other pro-inflammatory cells like neutrophils, eosinophils, macrophages and mast cells [21].

To date, the exact pathomechanisms of COPD are not yet elucidated. Cigarette smoking is a primary risk factor for the development of COPD, but only 20% of smokers actually develop the disease, suggesting that genetic predisposition plays a role [22]. However, understanding the impact of toxin-producing bacteria on cigarette-smoke induced inflammation might provide novel insights into the pathogenesis of smoking-related disease such as COPD. Therefore, we investigated the effects of concomitant *Staphylococcus aureus *Enterotoxin B (SEB) application on a well established mouse model of cigarette-smoke (CS) induced inflammation [23]. We evaluated inflammatory cells and their mediators in bronchoalveolar lavage (BAL) fluid and lung tissue, looked at systemic effects by measuring serum immunoglobulins, and evaluated goblet cell hyperplasia and lymphoid neogenesis.

## Methods

### Experimental protocol

**Figure 1:**
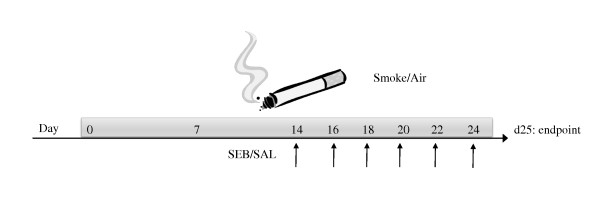
**Experimental protocol**. Male C57BL/6 mice (n = 8) were exposed to cigarette smoke(CS) of five cigarettes, four times per day with 30 min smoke-free intervals. Controls were exposed to air. Starting from day 14 of the CS exposure, mice received concomitant endonasal application of SEB (50 μL - 10 μg/mL) or saline, on alternate days.

Male C57BL/6 mice (n = 8), 6-8 weeks old were purchased from Charles River Laboratories (Brussels, Belgium). Mice were exposed to the tobacco smoke of five cigarettes (Reference Cigarette 2R4F without filter, University of Kentucky, Lexington, KY, USA) four times per day with 30 min smoke-free intervals [24]. The animals were exposed to mainstream cigarette smoke (CS) by whole body exposure, 5 days per week for 4 weeks. Control groups (8 age-matched male C57BL/6 mice) were exposed to air. Starting from day 14 of the CS exposure, mice received concomitant endonasal application of SEB (50 μL - 10 μg/mL - Sigma-Aldrich, LPS content below detection limit) or Saline, on alternate days. This dose was chosen based on Hellings *et al*. [18] For the application, mice were slightly anaesthetized with isoflurane, and six applications were performed as depicted in Figure 1. All experimental procedures were approved by the local ethical committee for animal experiments (Faculty of Medicine and Health Sciences, Ghent University). The results section contains data from one representative experiment out of three independent experiments.

### Bronchoalveolar lavage and cytospins

Twenty-four hours after the last cigarette smoke (CS) exposure and endonasal application, mice were sacrificed by a lethal dose of pentobarbital (Sanofi-Synthelabo). A cannula was inserted in the trachea, and BAL was performed by instillation of 3 × 300 μl of HBSS supplemented with BSA for cytokine measurements. Three additional instillations with 1 ml of HBSS plus EDTA were performed to achieve maximal recovery of BAL cells. A total cell count was performed in a Bürker chamber. Approximately fifty thousand BAL cells were processed for cytospins and were stained with May-Grünwald-Giemsa for differential cell counting. The remaining cells were used for FACS analysis.

### Preparation of lung single-cell suspensions

Blood was collected via retro-orbital bleeding. Then, the pulmonary and systemic circulation was rinsed to remove contaminating blood cells. Lungs were taken and digested as described previously [24]. Briefly, minced lung pieces were incubated with 1 mg/ml collagenase and 20 μg/ml DNase I for 45 min at 37°C. Red blood cells were lysed using ammonium chloride buffer. Finally, cell suspensions were filtered through a 50-μm nylon mesh to remove undigested organ fragments.

### Flow cytometry

All staining procedures were conducted in calcium- and magnesium-free PBS containing 10 mM EDTA, 1% BSA (Dade Behring), and 0.1% sodium azide. Cells were preincubated with anti-CD16/CD32 (2.4G2) to block Fc receptors. Antibodies used to identify mouse DC populations were anti-CD11c-allophycocyanin (APC; HL3) and anti-I-Ab-phycoerythrin (PE; AF6-120.1). The following mAbs were used to stain mouse T-cell subpopulations: anti-CD4-fluorescein isothiocyanate (FITC; GK1.5), anti-CD8-FITC (53-6.7), anti-CD3-APC (145-2C11) and anti-CD69-PE (H1.2F3). To identify granulocytes, anti-Gr-1-PE (RB6-8C5) and anti-CD11c-APC (HL3) were used. As a last step before analysis, cells were incubated with 7-aminoactinomycin D (or viaprobe; BD Pharmingen) for dead cell exclusion. All labeling reactions were performed on ice in FACS-EDTA buffer. Flow cytometry data acquisition was performed on a FACScalibur™ running CellQuest™ software (BD Biosciences, San Jose, CA, USA).

### Measurement of Immunoglobulins

Retro-orbital blood was drawn for measurement of total IgE, IgG, IgM and IgA with ELISA. Commercially available ELISA kits were used to determine serum and BAL titers of IgG (ZeptoMetrix, Buffalo, NY, USA), IgM (ZeptoMetrix, Buffalo, NY, USA) and IgA (Alpha Diagnostic International, San Antonio, TX, USA). For the measurement of total IgE, a two-side in-house sandwich ELISA was used, with two monoclonal rat anti-mouse IgE antibodies reacting with different epitopes on the epsilon heavy chain (H. Bazin, Experimental Immunology Unit, UCL, Brussels, Belgium). The second antibody was biotinylated and detected colorimetrically after adding horseradish peroxidase-streptavidine conjugate. Absorbance values, read at 492 nm (Labsystems Multiscan RC, Labsystems b.v., Brussels, Belgium) were converted to concentrations in serum and BAL fluid by comparison with a standard curve obtained with mouse IgE of known concentration (H. Bazin)

### Goblet cell analysis

Left lung was fixed in 4% paraformaldehyde and embedded in paraffin. Transversal sections of 3 μm were stained with periodic acid-Schiff (PAS) to identify goblet cells. Quantitative measurements of goblet cells were performed in the airways with a perimeter of basement membrane (Pbm) ranging from 800 to 2000 μm. Results are expressed as the number of goblet cells per millimeter of basement membrane.

### Morphometric quantification of lymphoid neogenesis

To evaluate the presence of lymphoid infiltrates in lung tissues, sections obtained from formalin-fixed, paraffin-embedded lung lobes were subjected to an immunohistological CD3/B220 double-staining as described previously [24]. Infiltrates in the proximity of airways and blood vessels were counted. Accumulations of ≥50 cells were defined as lymphoid aggregates. Counts were normalized for the number of bronchovascular bundles per lung section.

### RT-PCR analysis

Total lung RNA was extracted with the Rneasy Mini kit (Qiagen, Hilden, Germany). Expression of CXCL-13, CCL19, IL-13 and MIP-3α mRNA relative to HPRT mRNA [25], were performed with Assay-on-demand Gene Expression Products (Applied Biosystems, Foster City, CA, USA). Real-time RT PCR for CCL21-leucine and CCL21-serine started from 25 ng of cDNA. Primers and FAM/TAMRA probes were synthesized on demand (Sigma-Proligo). Primer/probe sequences and PCR conditions were performed as described previously [26,27].

### Protein measurement in BAL

CXCL13 protein levels in BAL supernatant were determined using a commercially available ELISA (R&D Systems, Abingdon, UK). Cytometric Bead Array (BD Biosciences, San Jose, CA, USA) was used to detect the cytokines KC, MCP-1, IL-17A and IFN-γ in the supernatant of BAL fluid.

### Statistical analysis

Reported values are expressed as mean ± SEM. Statistical analysis was performed with SPSS software (version 18.0) using nonparametric tests. The different experimental groups were compared by a Kruskal-Wallis test for multiple comparisons. When a p-value ≤ 0.05 was obtained with the Kruskal-Wallis test, pairwise comparisons were made by means of a Mann-Whitney U test with Bonferroni corrections for multiple comparisons. A p-value p ≤ 0.05 was considered significant.

## Results

### SEB aggravates the CS-induced pulmonary inflammation

To evaluate the effects of *Staphylococcus aureus *enterotoxin B (SEB) on cigarette smoke (CS)-induced pulmonary inflammation, C57Bl/6 mice were exposed to CS for 4 weeks, with a concomitant SEB exposure during the last 2 weeks (Figure 1).

**Figure 2:**
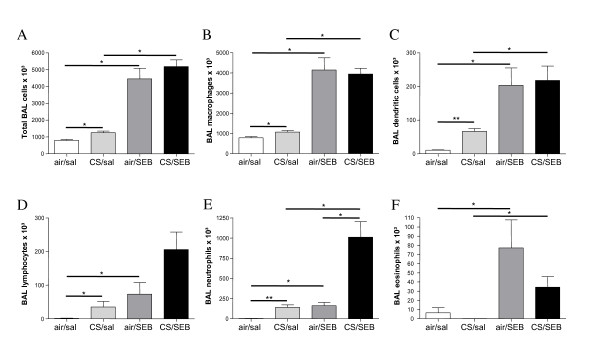
**BAL fluid analysis**. Total BAL cells and cell differentiation in BAL fluid of mice exposed to saline or SEB, combined with air or CS. A) Total BAL cells, B) macrophages, C) dendritic cells, D) lymphocytes, E) neutrophils, F) eosinophils. Results are expressed as mean ± SEM, n = 8 animals/group, *p < 0.05, **p < 0.01.

In BAL fluid, sole endonasal SEB application and sole CS-exposure resulted in increased numbers of total cells, alveolar macrophages, dendritic cells (DCs), lymphocytes and neutrophils, compared to air/saline exposed animals (Figure 2A-E). However, these increases in cell numbers were much more pronounced upon SEB application compared to CS-exposure. Also a modest eosinophilic inflammation was observed in the SEB-exposed groups (Figure 2F).

Interestingly, the combination of CS exposure and SEB significantly increased BAL neutrophil numbers compared to sole CS or SEB exposure (Figure 2E). Also BAL lymphocyte numbers in smoke-exposed mice were increased upon SEB application (Figure 2D).

**Figure 3:**
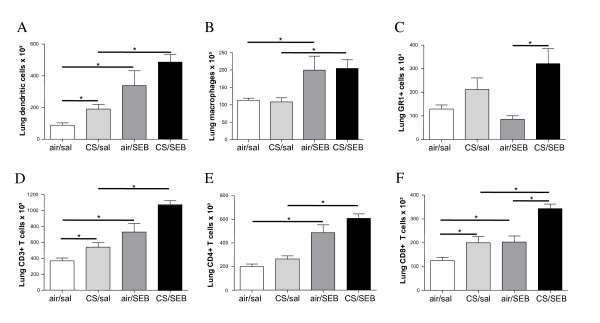
**Lung cell differentiation**. Flow cytometric analysis of cells from lung digest: A) dendritic cells, B) macrophages, C) GR1^+ ^cells, D) CD3^+ ^T lymphocytes, E) CD4^+ ^T lymphocytes and F) CD8^+ ^T lymphocytes from mice exposed to saline or SEB, combined with air or CS. Results are expressed as mean ± SEM, n = 8 animals/group, *p < 0.05.

In lung single cell suspensions, SEB solely induced an increase in DCs, CD3^+ ^T cells and macrophages, whereas CS exposure caused increased DCs and CD3^+ ^T cells in lung tissue (Figure 3A, D, B).

Interestingly, combined CS and SEB exposure caused a further increase in CD3^+ ^T cells, and more specifically CD8^+ ^T-cells, compared to CS or SEB alone (Figure 3D, F). Also DC, CD4^+ ^T-cells and GR1^+ ^cells tended to be higher in the combined CS/SEB group versus sole CS or SEB application (Figure 3A, E, C).

### Increased IL-17A in BAL upon combined SEB and CS exposure

**Figure 4:**
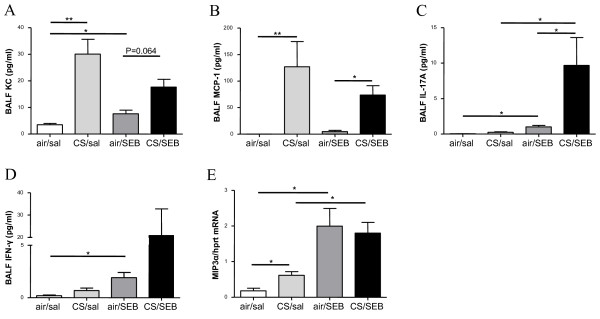
**Protein measurements in BAL fluid**. Protein levels of A) KC, B) MCP-1, C) IL-17A, D) IFN-γ in BAL fluid of mice exposed to saline or SEB, combined with air or CS, as measured with ELISA. E) mRNA expression of MIP-3α in total lung tissue, measured by RT-PCR. The results are expressed as ratio with hypoxanthine guanine phosphoribosyltransferase (HPRT) mRNA. Results are expressed as mean ± SEM, n = 8 animals/group, *p < 0.05, **p < 0.01.

As previously described [24], 4-wk CS-exposure clearly induced high levels of KC (mouse homolog for IL-8) and MCP-1 in BAL (Figure 4A, B). In contrast sole SEB application induced a modest increase in KC, and very low levels of IFN-γ and IL-17A (Figure 4A, D, C). Whereas the CS-induced KC and MCP-1 levels in BAL were not affected by an additional SEB exposure, the combined CS and SEB exposure did induce IL-17A levels in BAL, compared to single CS or SEB exposure (Figure 4C). Also IFN-γ levels tended to be highest in the combined CS/SEB group (Figure 4D).

mRNA levels of MIP-3α were increased after both CS or SEB exposure. Combined CS/SEB exposure did not cause a further MIP-3α increase (Figure 4E).

### SEB induces IgA and IgM levels in BAL

**Figure 5:**
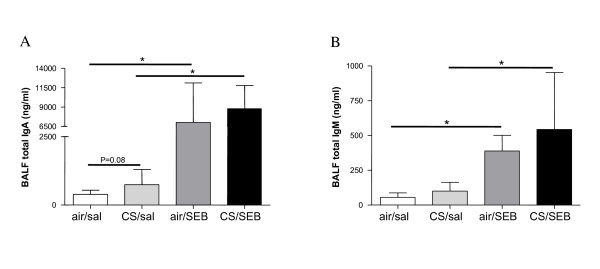
**BAL fluid immunoglobulin levels**. A) Total IgA and B) total IgM in BAL fluid of mice exposed to saline or SEB, combined with air or CS. Results are expressed as mean ± SEM, n = 8 animals/group, *p < 0.05.

Systemic effects of either CS or SEB, or both were evaluated in serum, but no significant differences in total IgG, IgM, IgA or IgE levels were detected between the experimental groups. In BAL, CS exposure tended to increase IgA. Both IgA and IgM levels in BAL were significantly increased upon SEB-exposure (Figure 5). IgE in BAL was below the detection limit.

### Combined CS/SEB exposure affects epithelial remodeling

**Figure 6:**
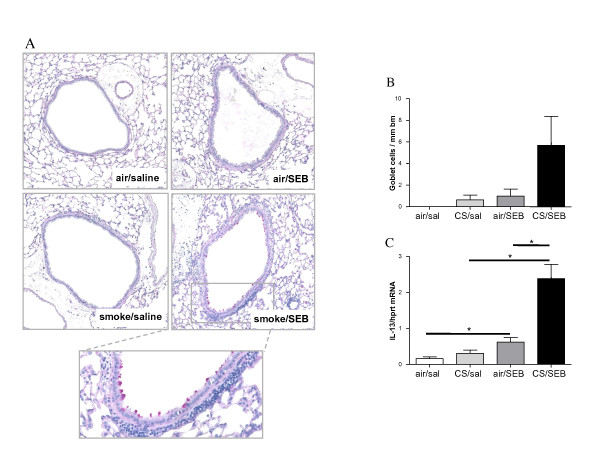
**Epithelial remodeling**. A) Histological evaluation of goblet cell hyperplasia on Periodic Acid Schiff (PAS) stained lung tissue sections of mice exposed to saline or SEB, combined with air or CS. B) Quantification of goblet cells. C) mRNA expression of IL-13, relative to a housekeeping gene (HPRT) was measured on total lung homogenates by RT-PCR. Results are expressed as mean ± SEM, n = 8 animals/group, *p < 0.05.

Epithelial remodeling was evaluated by counting the number of PAS-positive goblet cells per millimeter of basement membrane. A strong tendency towards increased numbers of goblet cells in the CS/SEB mice was observed, compared to all other conditions (Figure 6A, B). This finding correlated nicely with a significant increase in IL-13 mRNA expression in total lung in CS/SEB mice (Figure 6C).

### Combined CS/SEB induces the formation of dense lymphoid aggregates in lung tissue

**Figure 7:**
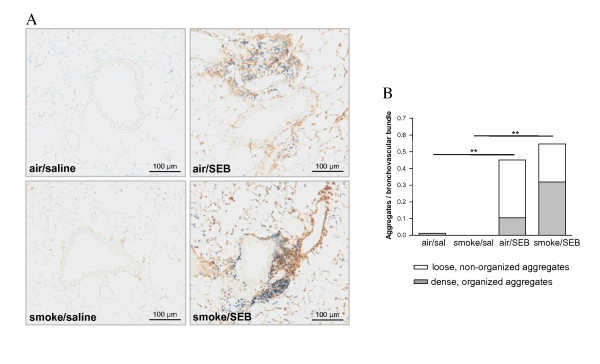
**Evaluation of lymphoid aggregates in lung tissue**. A) Photomicrographs of lymphoid aggregates in CD3/B220 immuno-stained lung tissue of mice exposed to saline or SEB, combined with air or CS (brown: CD3 positive cells; blue: B220 positive cells). B) Quantification of loose and dense lymphoid aggregates located in the bronchovascular area. Results are expressed as mean, n = 8 animals/group, *p < 0.05, **p < 0.01.

Previously, our group has demonstrated increased lymphoid neogenesis after 6 months of CS-exposure [25]. As earlier shown in the CS-model, subacute CS-exposure as such did not result in lymphoid neogenesis. Interestingly however, already after 4-wk CS-exposure, dense, organized lymphoid aggregates could be demonstrated in the combined CS/SEB group whereas air/SEB mice displayed mainly loose, non-organized lymphoid aggregates (Figure 7).

**Figure 8:**
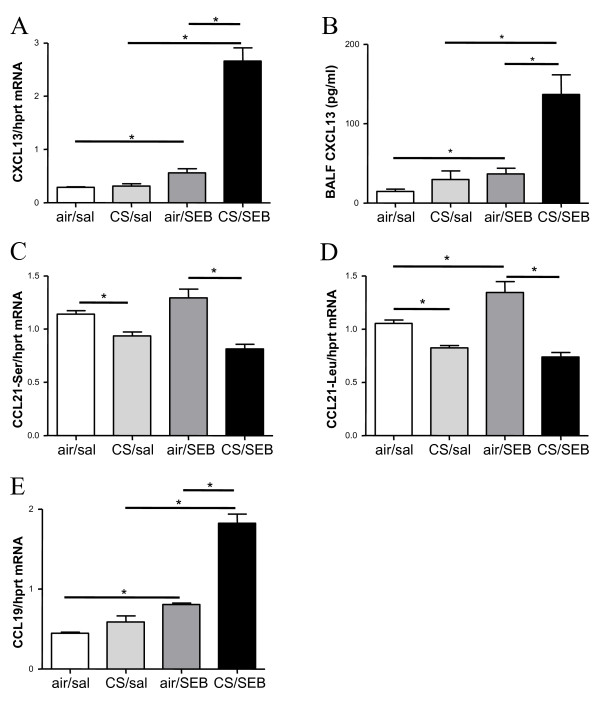
**Chemokines involved in the homeostatic trafficking of leukocytes**. Measurements of lymphoid chemokines in lung tissue and BAL fluid. mRNA expression of A) CXCL-13, C) CCL21-Ser, D) CCL21-Leu and E) CCL-19 in total lung tissue of mice exposed to saline or SEB, combined with air or CS, measured by RT-PCR. The results are expressed relative to HPRT mRNA. B) Protein levels of CXCL-13 in BAL fluid as measured by ELISA. Results are expressed as mean ± SEM, n = 8 animals/group, *p < 0.05.

Since CXCL13, CCL19 and CCL21 are chemokines involved in the homeostatic trafficking of leukocytes, mainly lymphocytes, to the secondary and tertiary lymphoid tissues, their expression was also evaluated in this model. The increase in dense lymphoid aggregates in CS/SEB mice correlated nicely with significant increases in CXCL13 (protein levels in BAL fluid, mRNA levels in total lung) (Figure 8A, B) and CCL19 (mRNA levels) expression in CS/SEB mice compared to all other groups (Figure 8E). CCL21 mRNA levels (both isoforms CCL21-Ser and CCL21-Leu) decreased upon CS exposure, confirming previous findings of CCL21 downregulation upon subacute CS exposure [26] and decreased even further in the CS/SEB group. Intriguingly, the CCL21 mRNA levels of both isoforms tended to increase upon sole SEB exposure (Figure 8C, D).

## Discussion

We hereby describe a novel mouse model of combined *Staphylococcus aureus *enterotoxin B (SEB) application and cigarette smoke exposure, which results in a significant aggravation of key features of CS-induced pulmonary inflammation, such as neutrophils and CD8^+ ^T cells in BAL and lung. Furthermore, levels of IL-17A in BAL were significantly increased upon concomitant SEB and CS exposure, compared to sole exposures of SEB or CS. In addition, tendencies of increased goblet cell hyperplasia, IL-13 mRNA expression and lymphoid neogenesis in smoke/SEB mice have been demonstrated, as well as increased expression of the relevant chemokines CXCL13 and CCL19. Altogether, these findings point to a possible disease-modifying role for SEB in CS-induced inflammation in this mouse model of subacute CS exposure.

Increasing evidence from human and murine research suggests that SEB is able to aggravate underlying disease. Moreover, SEB itself is also able to induce inflammation, depending on the dosage and timing of the experimental protocol [16,19]. Interestingly, these findings are not confined to SEB, as other staphylococcal superantigens demonstrate similar effects upon mucosal contact [28,29]. In line with previously reported findings, in our model sole endonasal SEB application caused an increase in total BAL cell number, lymphocytes and neutrophils [16]. Moreover, we could demonstrate raised numbers of macrophages and dendritic cells, a finding previously reported after *S. aureus *enterotoxin A exposure [28,29]. In the latter studies however, the authors could not demonstrate increased eosinophils, which was the case in our model. The superantigen effect of SEB caused the expected lymphocyte accumulation in BAL, which appeared to be non-specific, as both CD4^+ ^and CD8^+ ^T cells were increased. These data stress the potency of staphylococcal superantigens of initiating a massive immune response.

Concomitant CS/SEB exposure lead to a remarkable increase in neutrophil number, compared to CS or SEB exposure alone. Although the findings for neutrophils in lung (measured with granulocyte marker GR-1) were less convincing than in BAL, the combined CS/SEB group showed the highest number of GR-1^+ ^cells. Interestingly, also the CD8^+ ^T cell fraction in lung single cell suspensions, was significantly upregulated when smoke and SEB were combined. The potential clinical relevance of increased neutrophil and CD8^+ ^T-cell numbers lays in the fact that neutrophilic inflammation in the airways in smokers correlates with an accelerated decline in lung function [30], and increased T-cell numbers correlate with the amount of alveolar destruction and the severity of airflow obstruction [31].

We confirm an increased MIP-3α expression in lungs after CS exposure leading to an accumulation of dendritic cells in this model [24]. Interestingly, this increase in MIP-3α is also seen after SEB exposure, with raised DCs in BAL and airway parenchyma in these groups.

As previously demonstrated in the subacute CS-model, we have observed an increase in levels of KC and MCP-1 after 4-wk CS exposure [24], explaining the accumulation of inflammatory cells in BAL and lung. Sole SEB application on the other hand resulted in raised levels of KC, IFN-γ and IL-17A, but not MCP-1. Interestingly, the combined exposure of smoke and SEB further increased the IL-17A levels, which might explain the exacerbated BAL neutrophilia in CS/SEB mice. Indeed, IL-17 is known to be important in neutrophil maturation, migration and function in the lung tissue and airways. Furthermore, IL-17 induction of neutrophil activation and migration is important in defense against organisms infecting the lung [32]. Interestingly, IL-17 can also induce eosinophilic accumulation, in particular circumstances [33].

IL-17 is normally produced by CD4^+ ^T cells, although it might also arise from CD8^+ ^T cells and in some cases even from macrophages, neutrophils or eosinophils [34], as a necessary step in the normal immunity against bacterial infections in the airways. However, IL-17 has been linked to unfavorable outcome to infection, in particular in the presence of IFN-γ [35], resulting a high inflammatory pathology and tissue destruction. Increasing evidence dedicates a role to exaggerated recruitment and activation of neutrophils in the clinical course of airway diseases like COPD. Therefore, it is tempting to speculate on a role for SEB in the induction of IL-17 release, leading to the aggravation of cigarette smoke-induced inflammation, with increased number and activation of neutrophils, which causes amplification of tissue destruction and subsequent disease progression.

In addition, we could observe already after 4-wks an increase in the number of dense lymphoid aggregates in CS/SEB mice, linked to increased levels of CXCL13 and CCL19, which are attractants for B- and T-cells respectively. Moreover, it has been described that the respective receptors for these chemokines - CXCR5 and CCR7 - are also expressed on Th17 cells migrating into inflamed tissue [36], indicating a potential contribution of IL17-producing Th17 cells in this model of early COPD. The finding that lymphoid aggregates and the chemokines responsible for their neogenesis and organization [25] are already upregulated after 4-wk CS/SEB exposure, stresses the clinical relevance of this novel model of combined CS and enterotoxin exposure.

Staphylococcal superantigens are able to cause massive polyclonal T and B cell proliferation. Upon local application, as is done in this model, this leads to the mucosal synthesis of immunoglobulins, explaining the observed increase in BAL IgA and IgM. In humans, it is thought that continuous microbial stimulation leads to B cell turnover and plasma cell formation in nasal polyp disease, leading to an overproduction of immunoglobulins [37].

In this mouse model of early stage COPD with goblet cell hyperplasia and increased number of lymphoid follicles, endonasal SEB application has resulted in augmented CS-induced lower airway inflammation. CS and subsequent bacterial colonization are, amongst others, factors believed to determine both progression of COPD, as well as the frequency and severity of COPD exacerbations [38]. Therefore, mouse models of CS and bacterial co-exposure have been used in the past, mainly using *Haemophilus influenzae *[39]. Bacterial colonization and infection is rare in lower airways, but not in upper airways. Local carriage of enterotoxin-producing *S. aureus *in the nasal cavity is common, although multiple sites can be colonized (e.g. skin, pharynx and perineum) [40]. These toxins, like toxic shock syndrome toxin-1 (TSST-1), are known superantigens causing systemic diseases like food poisoning and toxic shock syndrome [4]. In nasal polyp disease, these toxins are believed to drive the local immunoglobulin production in response to enterotoxin-producing *S. aureus*.

The use of a single toxin instead of *S. aureus *in this model is both a strength and a limitation, since it simplifies the interpretation on one hand, but is not the real life situation on the other hand. Another limitation is that we cannot rule out endotoxin related effects in our model, although the LPS content of our SEB was below detection limit. Also the potential differences between our mouse model and the human situation concerning exposure to bacterial toxins and its effects on the balance of cytokines and inflammation is a limitation of the study. In addition, SEB on itself has resulted in pronounced inflammation in BAL and lungs, as it is a known superantigen. Finally, another possible limitation of this model is the short term (4-wk) CS exposure, whereas COPD is a chronic disease. Despite these limitations, altogether our findings indicate the importance of bacterial toxins present in the upper airways, affecting lower airway inflammation.

## Conclusion

The possible disease-modifying role for SAEs in COPD that has been described in humans [14], combined with our findings stress the potential role of airway colonizing and toxin-producing *Staphylococcus aureus*, in the pathophysiology of COPD [3].

## Competing interests

The authors declare that they have no competing interests.

## Authors' contributions

WH carried out the design and coordination of the study, gathered the data and interpreted the data, drafted and finalized the manuscript. EL gathered the data and interpreted the data, drafted and revised the manuscript. OK gathered the data and was involved in the critical reading of the manuscript. TD helped to optimize the PCR analyses for CXCL13 and CCL19. KB, PH, GB, GJ and CB were involved in the coordination and design of the study as well as the critical reading of the manuscript. TM participated in the coordination of the study, helped to interpret the data and critically revised the manuscript. All authors read and approved the final version of the manuscript.

## Acknowledgements

The authors would like to thank Greet Barbier, Eliane Castrique, Indra De Borle, Philippe De Gryze, Katleen De Saedeleer, Anouck Goethals, Marie-Rose Mouton, Ann Neessen, Christelle Snauwaert and Evelyn Spruyt for their technical assistance.

This project is supported by the Fund for Scientific Research - Flanders (FWO-Vlaanderen - Project G.0052.06), by a grant from the Ghent University (BOF/GOA 01251504), by the Interuniversity Attraction Poles program (IUAP) - Belgian state - Belgian Science Policy P6/35, and by grants to CB from the Fund for Scientific Research - Flanders, FWO, no. A12/5-HB-KH3 and G.0436.04, and to KB as a postdoctoral fellow of the Fund for Scientific Research Flanders (FWO).
